# Common elements for uncommon light: vector beams with GRIN lenses

**DOI:** 10.1038/s41377-019-0228-9

**Published:** 2019-11-29

**Authors:** Andrew Forbes

**Affiliations:** School of Physics, University of the Witwatersrand, Johannesburg, South Africa

**Keywords:** Transformation optics, Optical physics

## Abstract

A well-known defect introduced during the fabrication of GRIN lenses can be exploited for the creation, detection and wave-guiding of exotic forms of vectorial structured light, bringing the toolkit into the realm of common laboratory optics.

Many of today’s optical applications require customized light fields, so-called structured light, tailored in phase, amplitude and polarization. A highly topical class of structured light is that of vector beams with spatially inhomogeneous polarization structures, for example, the well-known cylindrical vector vortex beams (the natural modes of many optical fibers). These complex light fields have facilitated tremendous advances in communication, imaging, laser materials processing, quantum processes, and microscopy^1,2^. Creating these vector fields in the laboratory often requires custom optical elements such as liquid crystal devices and meta-surfaces: uncommon elements for uncommon light. In a recently published paper^3^, Martin Booth and collaborators demonstrated that common gradient index (GRIN) lenses, ubiquitous to most optical laboratories, can be harnessed for vectorial control of light, ushering in a simpler approach for the creation, detection and wave-guiding of these structured light fields (Fig. 1).

**Fig. 1:**
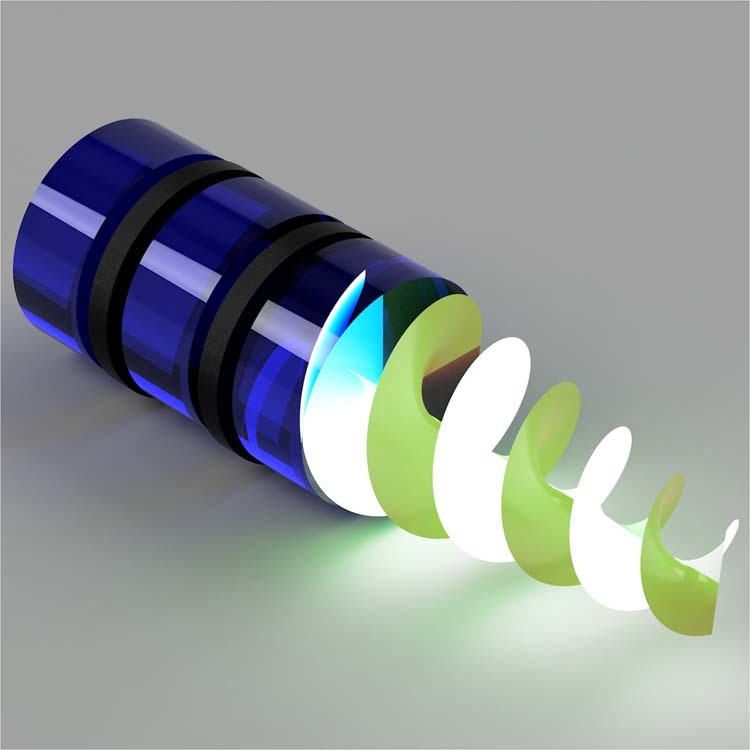
GRIN beam shaping. A cascade of GRIN lenses (blue) with other polarization modulating optics (black) sandwiched in between results in exotic forms of vectorial structured light carrying phase and polarization vortices. Similar configurations can be used to deliver tightly focused beams and to collect light for fast polarimetry.

GRIN lenses have a radially varying refractive index profile, allowing compact solutions for the guiding and focusing of light. In creating the rotationally symmetric refractive index profile, the ion-exchange process also introduces a symmetric birefringence. This process has been known for a long time and reluctantly accepted as a byproduct of the fabrication but is now shown to be useful, harnessed for control of custom optical fields. The combination of the dynamic phase from the refractive index profile and the geometric phase from the induced spatially varying birefringence means that light can be controlled in a more sophisticated manner than previously thought. The researchers used single and multiple GRIN lenses in a cascade that sandwiched between them other polarization controlling elements to demonstrate exotic states of vector light fields. In particular, the output light could contain both phase and polarization vortices^2^ due to the transfer of angular momentum to the fields by the inherent Pancharatnam-Berry phase of the GRIN lens. What is remarkable is that this control is achieved with a very common and inexpensive optical element.

To demonstrate the versatility of these devices, the team used single GRIN lenses to produce optical vortices and then cascades of elements to produce higher-order Poincare sphere beams. Of course, the GRIN lens is still a lens; in an exciting insight, the team showed that combining the two phase control mechanisms allowed for even better focusing performance. In particular, the team realized that until now, the input polarization state to GRIN lenses was not considered with due care, resulting in axial smearing about the focus. In fact, the team showed that the natural modes of the GRIN lens are particular vector vortex beams (radially and azimuthally polarized), each “seeing” its own refractive index profile, resulting in two apparent focusing abilities. While a combination of the two would smear the focus, a polarization eigenmode as the input would not: improved axial resolution in focusing can be achieved by structuring the GRIN lens input. The idea that the performance of simple GRIN lenses can be enhanced by polarization structuring the input light is a new paradigm for the optical community, with vast implications. A final demonstration exploited the reciprocity of light: rather than delivering or engineering light fields, the GRIN cascades could receive light for fast and compact polarimetry, unraveling the polarization properties of materials and light. This all-in-one approach will no doubt facilitate fast adoption by the broader community.

The results obtained by the team open the exciting possibility of a readily available structured light toolkit that can both create and deliver exotic states of light while simultaneously offering a compact device for fast polarimetry. Immediate applications are diverse and include improved imaging and analysis of biological samples and the optimal coupling of hybrid spin-orbit states into an optical fiber in classical and quantum communication.

Despite these exciting advances, there is still work to be done. The induced birefringence is small and highly variable from element to element; thus, in practice, a large cascade of elements may be needed for some applications. This problem could be overcome by deliberately inducing more of this “unwanted” side-effect in the fabrication process. The very concept of wave-guiding with geometric phase elements is still in its infancy^4^ and yet to be fully exploited, perhaps ushering in new functionality in GRIN optical devices. Finally, there is the possibility of combining vector beam generation by refraction in glass cones^5^ with further delivery and control in these GRIN systems for a monolithic solution in bulk glass. These exciting prospects are surely worthy of further attention from the community.
